# Asynchronous Distance Learning of the National Institutes of Health Stroke Scale During the COVID-19 Pandemic (E-Learning vs Video): Randomized Controlled Trial

**DOI:** 10.2196/23594

**Published:** 2021-01-15

**Authors:** Mélanie Suppan, Loric Stuby, Emmanuel Carrera, Philippe Cottet, Avinash Koka, Frédéric Assal, Georges Louis Savoldelli, Laurent Suppan

**Affiliations:** 1 Division of Anaesthesiology Department of Anaesthesiology, Clinical Pharmacology, Intensive Care and Emergency Medicine Geneva University Hospitals and Faculty of Medicine Geneva Switzerland; 2 Genève TEAM Ambulances Geneva Switzerland; 3 Stroke Center Department of Neurology Geneva University Hospitals and Faculty of Medicine University of Geneva Geneva Switzerland; 4 Division of Emergency Medicine Department of Anaesthesiology, Clinical Pharmacology, Intensive Care and Emergency Medicine Geneva University Hospitals and Faculty of Medicine Geneva Switzerland; 5 Cognitive Neurology Unit Neurology, Department of Clinical Neuroscience Geneva University Hospital and Faculty of Medicine Geneva Switzerland; 6 Unit of Development and Research in Medical Education Faculty of Medicine University of Geneva Geneva Switzerland

**Keywords:** stroke, COVID-19, e-learning, medical student, medical education, online learning, randomized controlled trial, video

## Abstract

**Background:**

The COVID-19 pandemic has considerably altered the regular medical education curriculum while increasing the need for health care professionals. Senior medical students are being incrementally deployed to the front line to address the shortage of certified physicians. These students, some of whom will be fast-tracked as physicians, may lack knowledge regarding the initial management of time-critical emergencies such as stroke.

**Objective:**

Our aim was to determine whether an e-learning module could improve asynchronous distance knowledge acquisition of the National Institutes of Health Stroke Scale (NIHSS) in senior medical students compared to the traditional didactic video.

**Methods:**

A randomized, data analyst–blinded web-based trial was conducted at the University of Geneva Faculty of Medicine between April and June 2020. Fifth year medical students followed a distance learning path designed to teach the NIHSS. The control group followed the traditional didactic video created by Patrick Lyden, while the e-learning group followed the updated version of a previously tested, highly interactive e-learning module. The main outcome was the score on a 50-question quiz displayed upon completion of the learning material. The difference in the proportion of correct answers for each specific NIHSS item was also assessed.

**Results:**

Out of 158 potential participants, 88 started their allocated learning path and 75 completed the trial. Participants who followed the e-learning module performed better than those who followed the video (38 correct answers, 95% CI 37-39, vs 35 correct answers, 95% CI 34-36, *P*<.001). Participants in the e-learning group scored better on five elements than the video group: key NIHSS concepts (*P*=.02), the consciousness – global item (*P*<.001), the facial palsy item (*P*=.04), the ataxia item (*P*=.03), and the sensory item (*P*=.04).

**Conclusions:**

Compared to the traditional didactic video, a highly interactive e-learning module enhances asynchronous distance learning and NIHSS knowledge acquisition in senior medical students.

## Introduction

The swift strengthening of public health policies in the context of the COVID-19 crisis has wrought deep changes in the regular medical education curricula of many countries [1-4] while also increasing the need for health care professionals, including physicians. Senior medical students are being incrementally used on the front lines to address the shortage of these professionals [5,6], and other students may soon be required to follow suit [7]. Accelerated graduation procedures have also been described in some regions [8]. Senior medical students as well as some of these fast-tracked physicians may lack knowledge regarding the initial management of specific emergencies such as stroke. Stroke is a time-critical emergency that must be treated swiftly to improve functional and vital prognoses [9]; however, disruptions in acute stroke pathways have been described in the wake of the COVID-19 pandemic [10]. The National Institutes of Health Stroke Scale (NIHSS) is widely used to assess stroke victims [11], and senior medical students as well as junior residents should be familiar with its application.

Traditional classroom or bedside teaching can be difficult to conduct in certain situations, such as a pandemic [12-14]. Many universities have strived to increase distance learning capabilities, thereby highlighting the potential benefits of electronic learning (e-learning) [15-17]. E-learning is a generic term that includes many types of technologically enhanced learning materials [18-20]. Asynchronous distance learning using these methods has yielded mixed results, probably due to differences in the quality of the content and the mode of delivery [21].

Since the release of Patrick Lyden’s didactic video in 1994 [22], the development of NIHSS teaching material has been rather limited. We have recently shown that compared to this didactic video, a highly interactive e-learning module improved NIHSS knowledge acquisition in paramedics [23]. We defined this module as “highly interactive” because it uses multiple learning mechanics to promote interaction and engagement. Among these mechanics, preventing content skipping [24] and providing feedback tailored to the user's answer were the most prominent [25]. Branching logic was extensively used to create this feedback.

This first study was performed with the participants present at the study site; therefore, they could immediately access technical support if needed. Moreover, although most results favored the use of the e-learning module, the control group was better at scoring the ataxia element than the e-learning group. Although video extracts were used within the e-learning module to demonstrate the assessment of almost all NIHSS items, the chapter regarding the ataxia element did not contain any video extracts. We therefore hypothesized that systematically embedding videos could improve NIHSS learning acquisition, and we updated the module accordingly.

Given the need for social distancing during the COVID-19 pandemic, our goal was to compare medical students’ asynchronous distance learning of the NIHSS using two different teaching tools: the gold standard didactic video and the updated version of our e-learning module.

## Methods

### Study Design and Setting

We performed a randomized, controlled, data analyst–blinded, web-based trial following the CONSORT-EHEALTH guidelines and incorporating relevant elements from the Checklist for Reporting Results of Internet E-Surveys (CHERRIES) checklist [26,27]. The study took place between April 28 and June 8, 2020, in Geneva, Switzerland. Fifth year medical students at the University of Geneva Faculty of Medicine (UGFM) were invited to take part in this trial on a voluntary basis.

### Standard Protocol Approvals, Registrations, and Participant Consent

Because the study included no patients and as no health outcomes were recorded, trial registration was not required according to the International Committee of Medical Journal Editors guidelines. Although the participants were not part of a vulnerable group according to Swiss federal law on human research [28], we filed a jurisdictional enquiry, and the regional ethics committee issued a “Declaration of no objection” (Req 2020-00474). The study was also approved by the Board of the Teaching Committee of the UGFM. Informed consent was gathered electronically.

### Enrollment

After gathering the necessary authorizations, the UGFM students’ secretary transmitted the exact number of fifth year medical students to MS, who performed a 1:1 computer assisted randomization without having access to any other data regarding the students. MS then created specific identifiers that were transmitted back to the UGFM along with a mailing template Multimedia Appendix 1. The UGFM staff were therefore unable to determine the students’ allocations or results. In addition, we were prevented from determining the students’ identities.

The students were informed of the goals of the investigation, were given information regarding data security and anonymization procedures, and were supplied with the email addresses of three investigators to allow them to ask further questions. Students who elected to browse the website were provided with additional information as well as with a link to a full 4-page consent form in PDF format that they could either print or save. Using their identifiers to log into the site was considered as acceptance to participate in the study. All participants were free to withdraw at any time. No financial incentive was provided.

### Web-Based Platform and Learning Material

We created a specific web-based platform under the Joomla 3.9 content management system (Open Source Matters) [29]. The control was Patrick Lyden’s original video, which was subtitled in French [22]. The experiment used version 21 of our e-learning module, which was developed using Articulate Storyline 3 (Articulate Global). This software enables the creation of many types of interactive content, including gamified modules and serious games [30,31], which can be accessed on regular computers as well as on smartphones and tablets.

**Figure 1:**
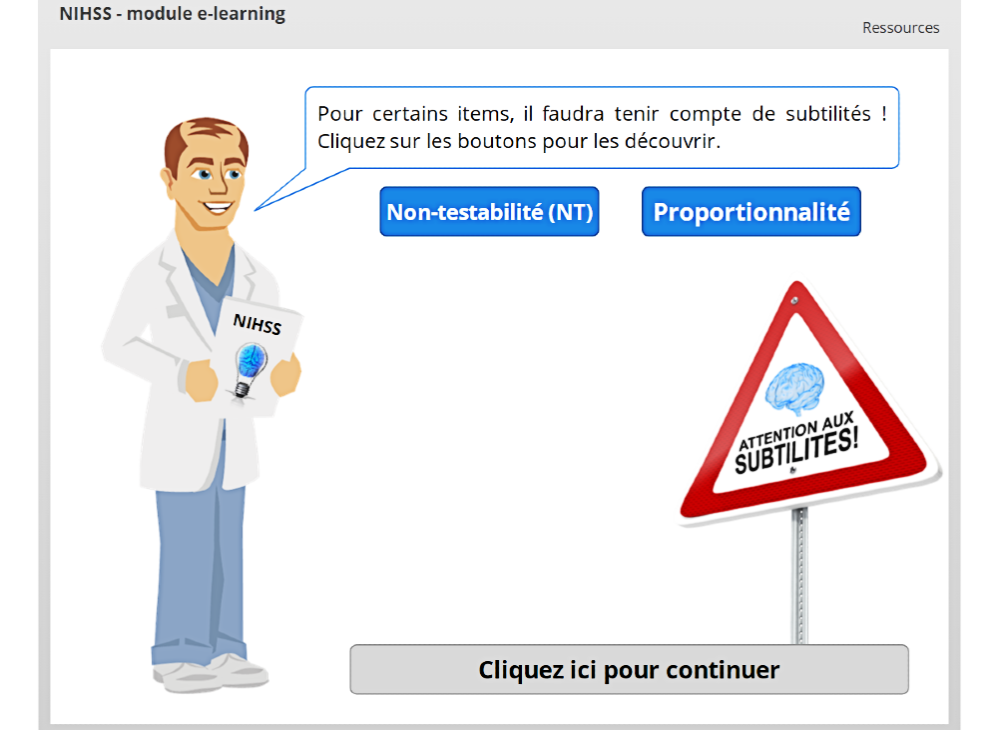
Prevention of content skipping. The user cannot click on the *Cliquez ici pour continuer* (Click here to continue) button until both blue buttons have been clicked.

**Figure 2:**
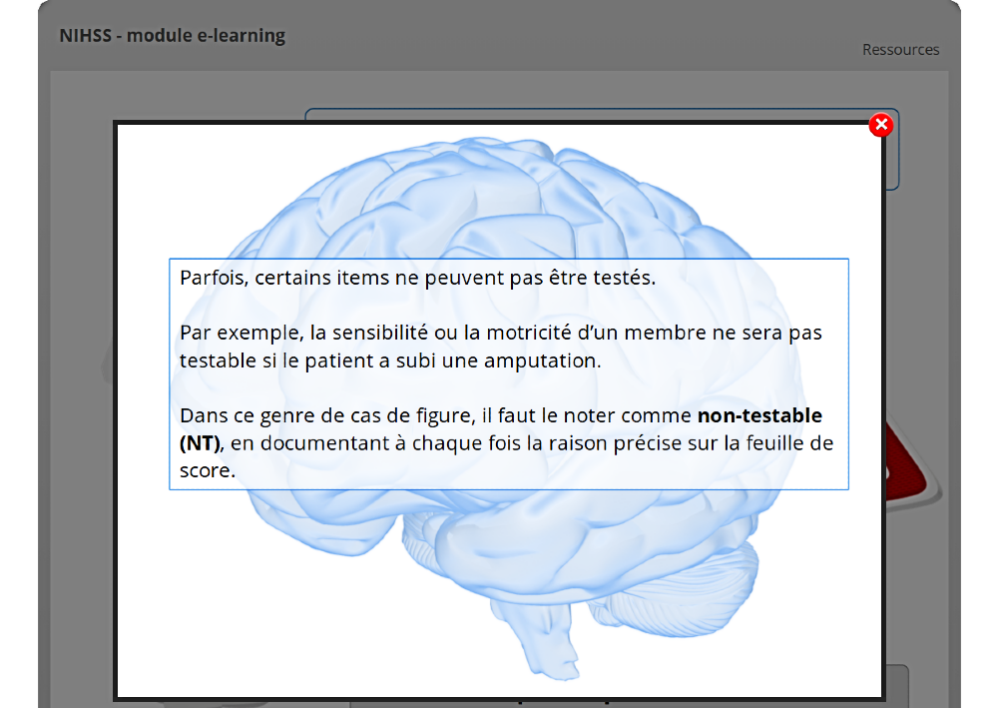
The user has clicked on one of the two buttons, and the learning content is now displayed in a lightbox. The *Cliquez ici pour continuer* (Click here to continue) button, which is slightly visible in the background, is still grey; therefore, it is inactive.

**Figure 3:**
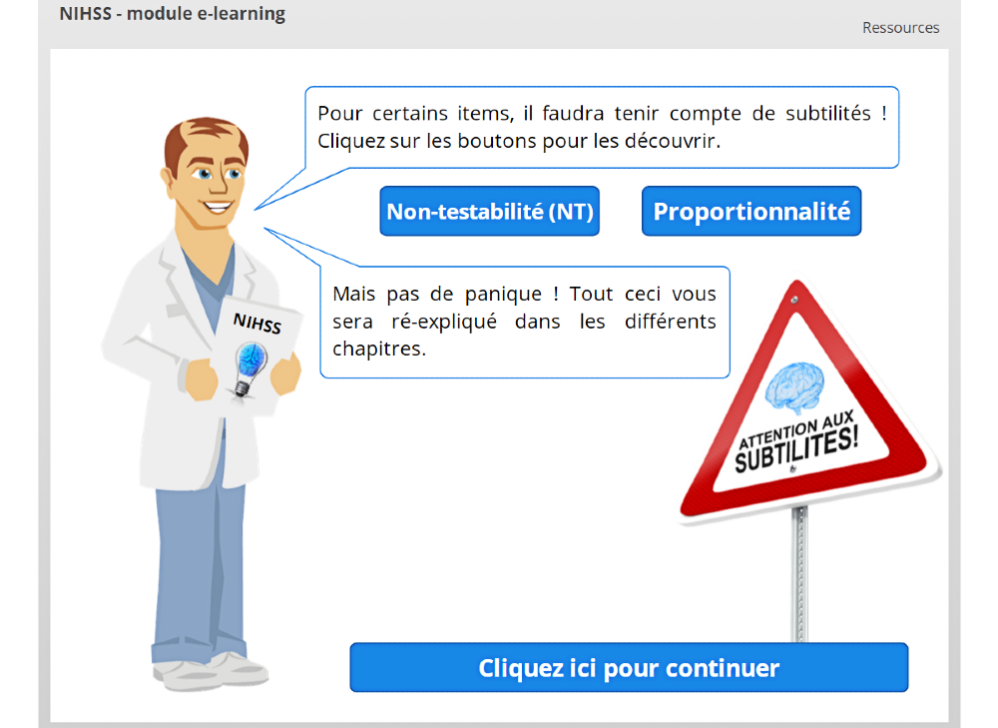
Both buttons have been clicked, and the user has seen both lightboxed slides. The *Cliquez ici pour continuer* (Click here to continue) button has thus been activated and is now colored blue.

The e-learning module contains 16 independent chapters. The first chapter is the introduction, which is automatically displayed when the module is launched. Prevention of content skipping is the first learning mechanic used in the module, and it already appears in the introductory slides (Figures 1-3) [24].

**Figure 4:**
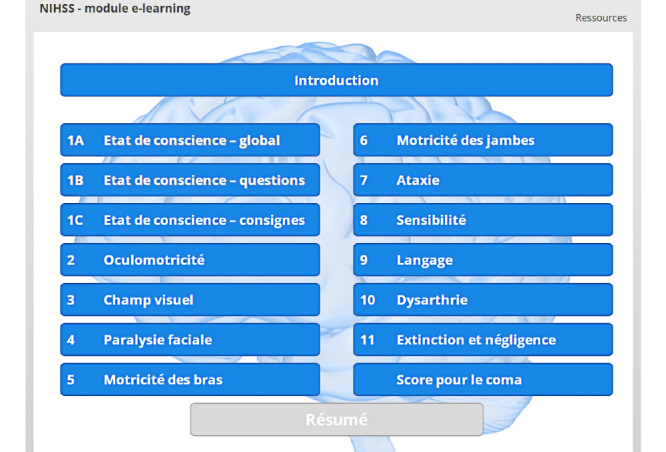
Table of contents of the e-learning module. The *Résumé* (Summary) can only be accessed once all the other chapters have been completed.

A table of contents is displayed as soon as the user has completed the introduction. The user can then choose to review the introduction or to access any other chapter apart from the summary (Figure 4).

Thirteen chapters are used to explain each specific NIHSS item (3 chapters are used to cover the first item, “consciousness,” which is divided into 3 elements). Although the chapters are numbered consecutively according to the NIHSS scoring logic, the user can freely elect the order in which to follow the chapters.

**Figure 5:**
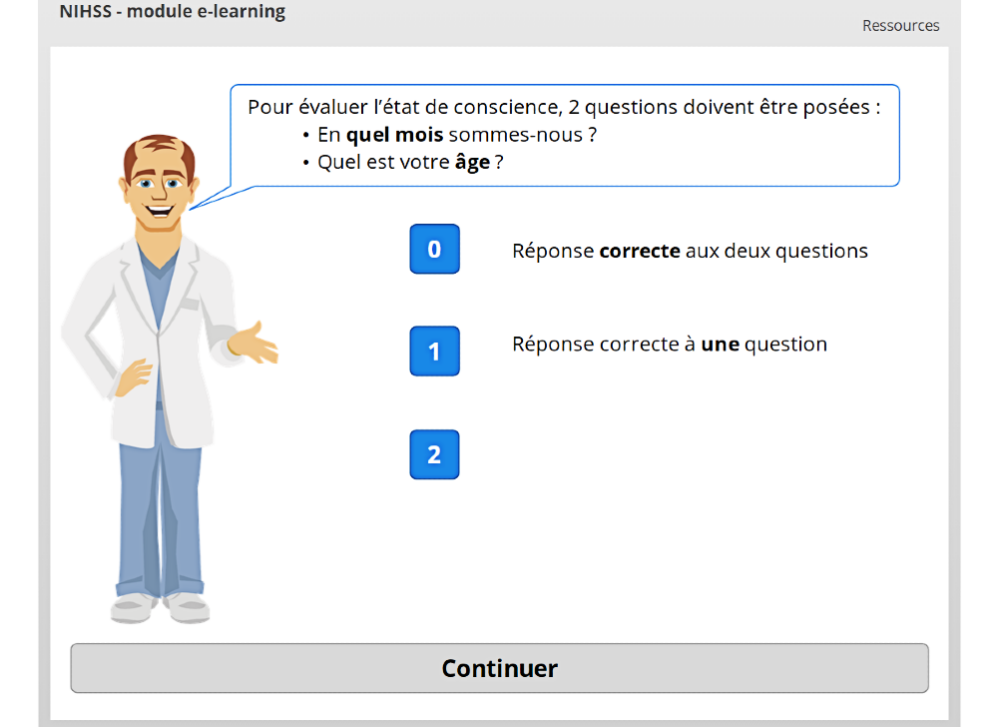
Prevention of content skipping. The user cannot click on the *Continuer* (Continue) button until all the blue buttons have been clicked.

All chapters include at least two learning mechanics. First, each chapter begins by displaying the NIHSS score specific to the scoring item, and users are once again prevented from skipping content, as they must click on each numbered button to discover the score (Figure 5).

**Figure 6:**
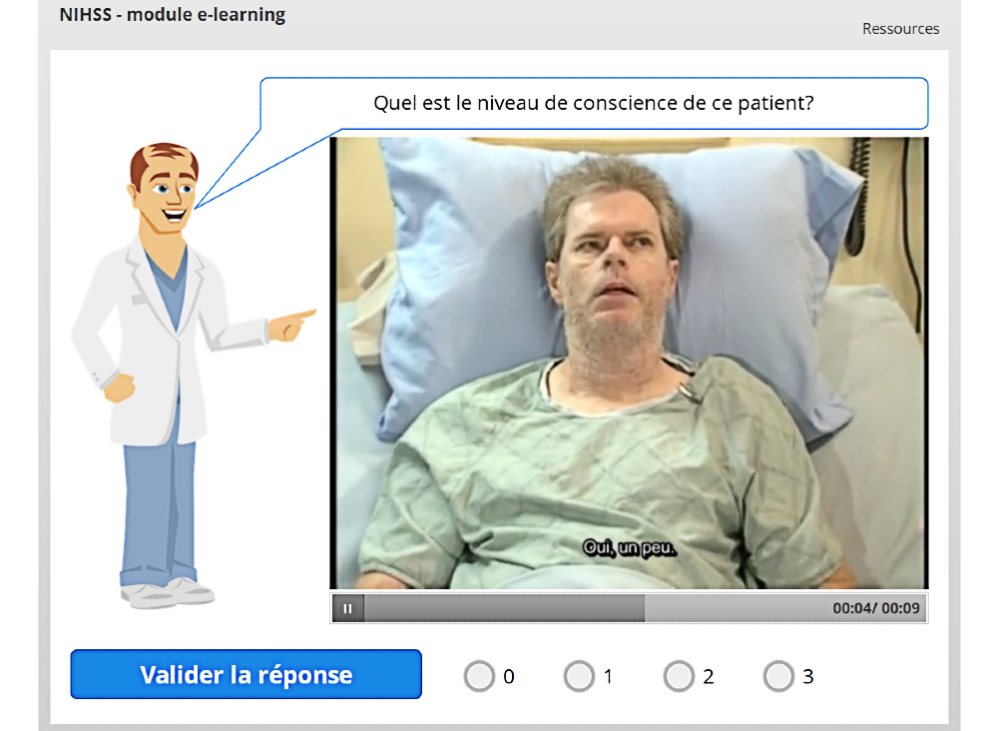
Video-based question. The user must choose the correct score for the patient displayed in the video before clicking on *Valider la réponse* (Validate the answer).

The second learning mechanic is linked to the use of subtitled videos. Video extracts are shown to the user, who must correctly score the NIHSS item (Figure 6). This version of the module contains video extracts in all chapters, including for the items related to dysarthria, level of consciousness – global, and level of consciousness – questions. In the previously studied version of our module (version 20), there were no video extracts for these items [23].

**Figure 7:**
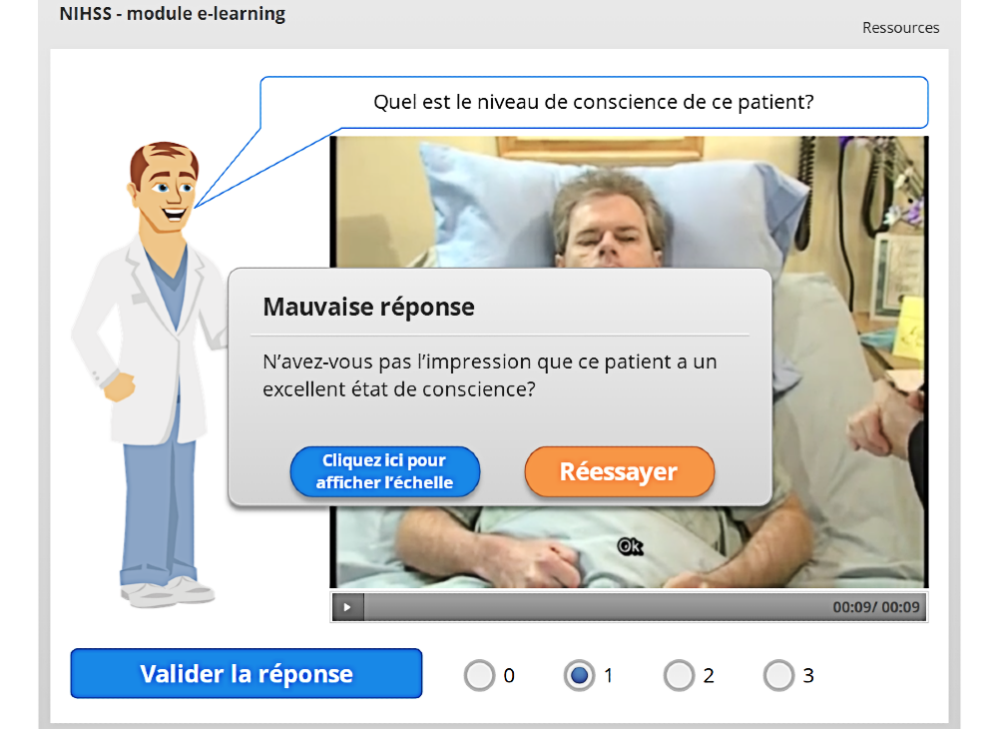
Wrong answer feedback and clue. The user can choose either to try again (*Réessayer*) or to review the scoring specific to this item by clicking *Cliquez ici pour afficher l’échelle* (Click here to display the scale).

Feedback is provided for each question [25]: if the answer is incorrect, a clue is given (Figure 7), and the user has the opportunity to review the NIHSS item scoring.

**Figure 8:**
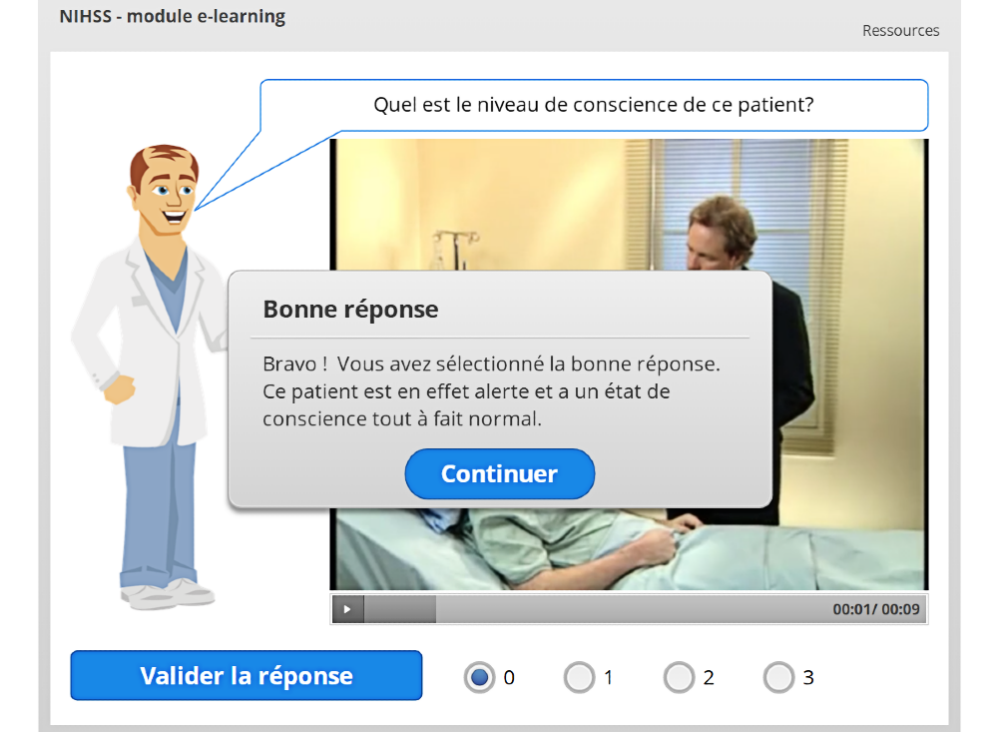
Positive feedback after a correct answer.

If the answer was correct, feedback is also given to reinforce the message (Figure 8).

**Figure 9:**
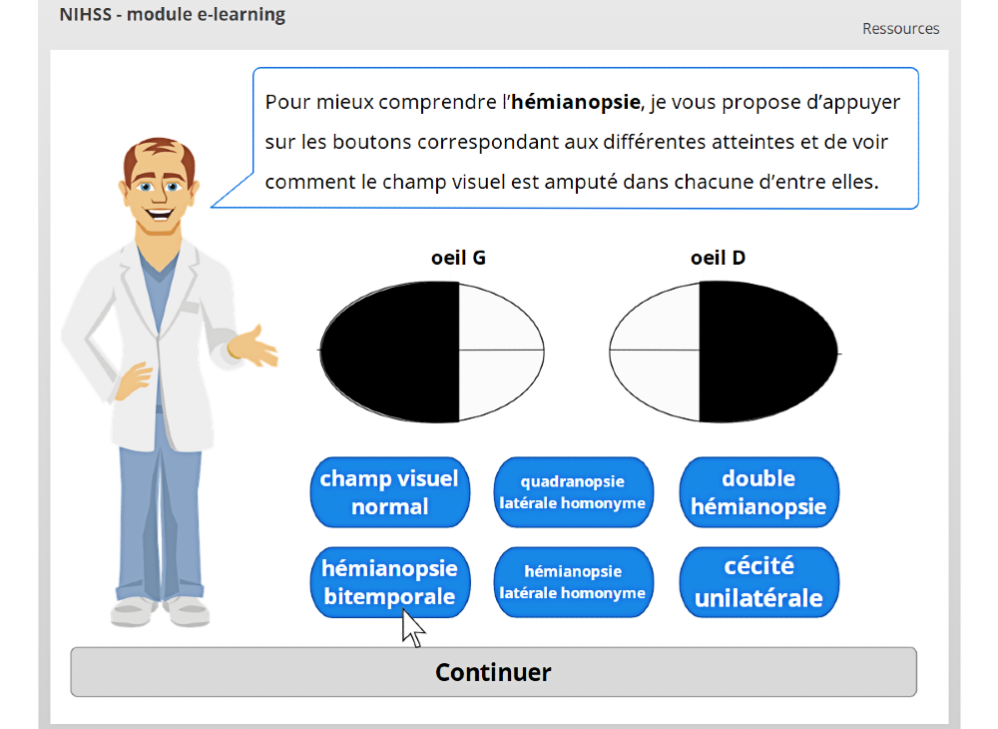
Interactive explanation of visual field deficit.

**Figure 10:**
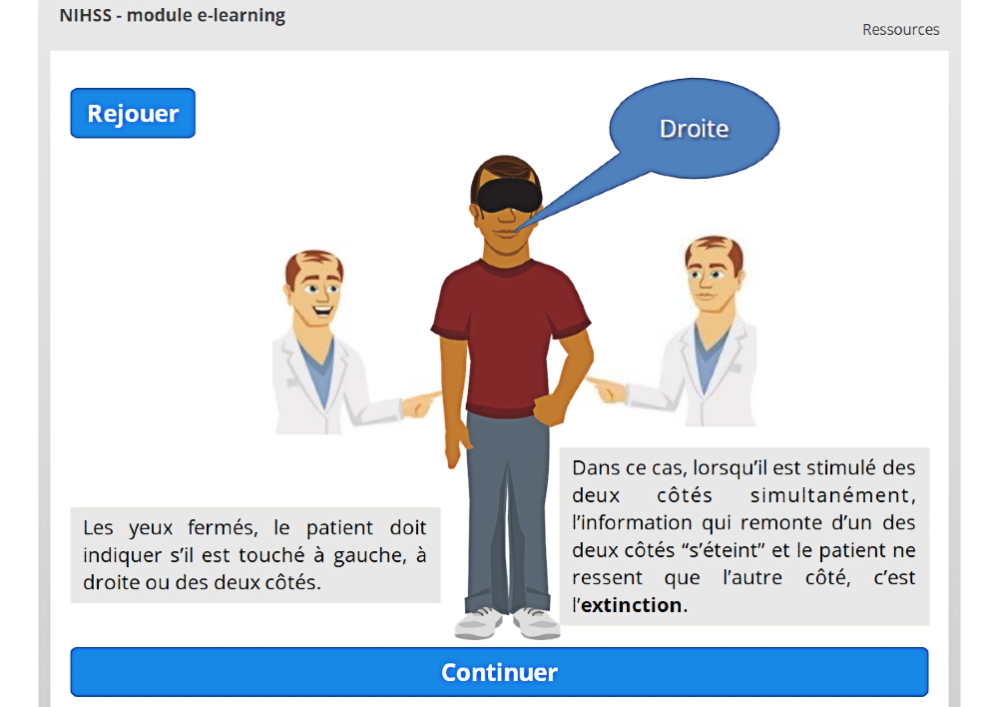
Animation used to explain extinction and inattention.

Specific interactions were designed to further illustrate particular elements, such as visual field deficits (Figure 9) or extinction and inattention (Figure 10).

The user can choose to exit the module at any time, as a prompt will be shown to allow the user to either resume the module or reset it. Before the summary chapter can be activated, users must complete a 14th chapter, which details the “coma score.”

This e-learning module, along with its previously studied iteration, can be accessed freely on the internet [32].

### Study Sequence

Immediately after login, the medical students learned which group they had been allocated to and were asked a first set of 6 questions displayed over a single page. Upon answering these questions, they could then access the learning material. No time limit was applied apart from the study end date (June 8, 2020). Once the learning material was completed, students were allowed to proceed to a 50-question quiz. This quiz was identical for all participants and contained five questions related to basic NIHSS concepts, followed by the clinical evaluation of 3 patients taken from Patrick Lyden’s certification videos. The NIHSS elements were displayed and scored in sequence according to the NIHSS scoring logic. After finalizing the quiz, participants were given their overall score as well as the possibility to review all questions at will and were shown their answers along with the correct answers. Then, 4 questions, based on a 5-point Likert scale, were asked to assess secondary outcomes, such as satisfaction. Students were finally given access to both the video and the e-learning module to discover the other teaching modality and/or to review the one they had just followed.

### Outcomes

The primary outcome of the study was the proportion of correct answers to the 50-question quiz. Secondary outcomes were the proportion of correct answers for each specific NIHSS item, user satisfaction, perceived adequacy of the time needed to complete the course, perceived difficulty of the course, probability that the participant would recommend the course, and whether the learning path had been completed over multiple days.

### Data Collection and Curation

Data were securely stored on an encrypted MariaDB 5.5.5 database (MariaDB Foundation) located on a Swiss server before being extracted in comma-separated values (CSV) format. We used STATA (StataCorp) for data curation and anonymization.

### Statistical Analysis

STATA 15.1 was used by L Stuby for statistical analysis. Incomplete answers to the 50-question quiz were not analyzed.

Normality was assessed by graphical evaluation and, if in doubt, we used the Shapiro-Wilk test. We applied the Fisher exact test to categorical variables and the Student *t* test or the Mann-Whitney U test to continuous variables according to normality. We considered a 2-sided *P* value <.05 as significant.

We used a convenience sample and calculated the power post hoc. We defined 4 sensitivity analyses a priori according to whether the participant had prior knowledge of the NIHSS, had already followed a specific NIHSS course, had worked in either an intensive care unit or in a neurology or neurosurgery ward for more than 3 months, or had completed the learning path over multiple days. This was defined as more than 12 hours elapsed between initiation and completion of the course.

Finally, we performed univariate followed by multivariable linear regression to look for possible confounding factors.

### Data Availability

Our curated data file is available on Mendeley Data [33].

## Results

**Figure 11:**
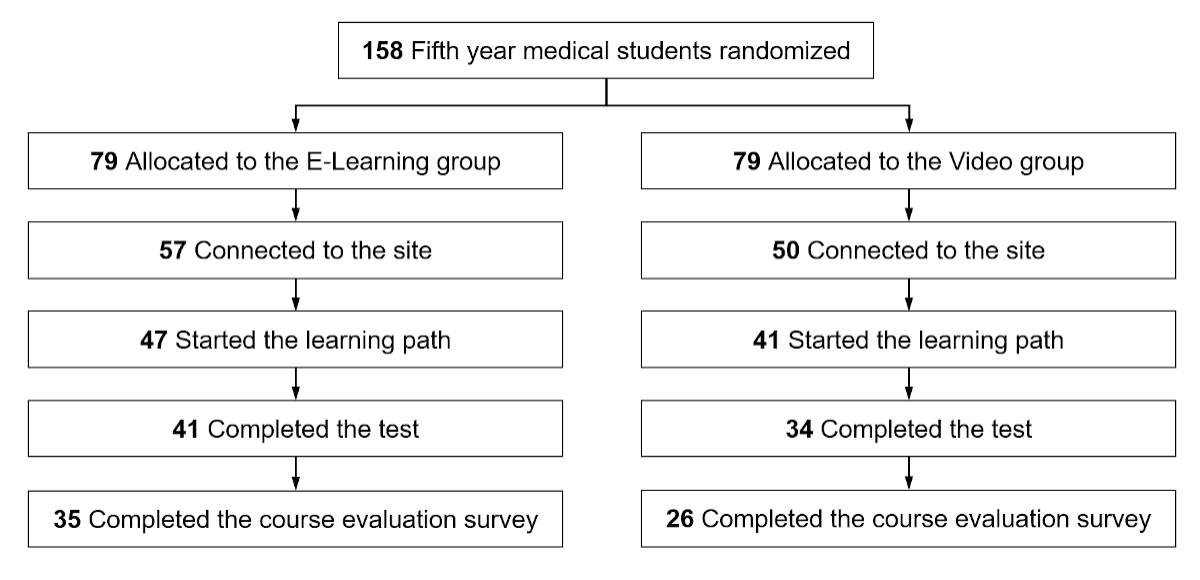
Study flowchart.

Out of 158 potential participants, 75 (47.5%) completed the trial (Figure 11). Table 1 details their characteristics.

After the first mailing (April 28, 2020), 21 students completed the trial. The first reminder (May 11, 2020) led 29 more students to complete the course, while another 25 participated after the second and last reminder (May 18, 2020).

**Table 1:** Participant data (N=75).

Characteristic	Value	
	Video group (n=34)	E-Learning group (n=41)
Age, median (Q1-Q3)	24 (23-25)	24 (23-24)
Prior knowledge of NIHSS^a^ application, n (%)	3 (9)	3 (7)
Specific NIHSS course followed, n (%)	4 (12)	2 (5)
E-learning NIHSS course followed, n (%)	0 (0)	0 (0)
Had worked in intensive care unit or neurology ward, n (%)	0 (0)	1 (2)
Missing data, n (%)	2 (6)	2 (5)

^a^NIHSS: National Institutes of Health Stroke Scale.

Participants who followed the e-learning module performed better than those who followed the video (38 correct answers, 95% CI 37-39, vs 35, 95% CI 34-36, *P*<.001). Participants in the e-learning group scored better on five elements than the video group: key NIHSS concepts (*P*=.02), the consciousness – global item (*P*<.001), the facial palsy item (*P*=.04), the ataxia item (*P*=.03) and the sensory item (*P*=.04). There was no such effect in the video group. Detailed results are shown in Table 2.

**Figure 12:**
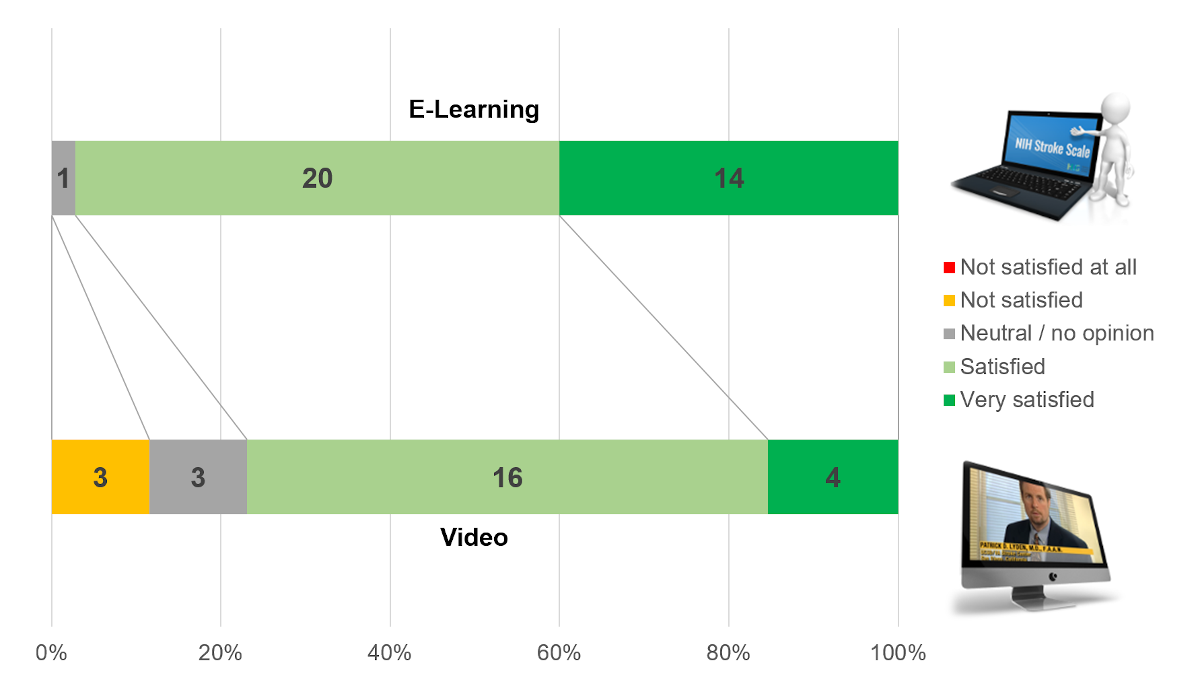
Satisfaction of the participants in the e-learning and video groups regarding the learning method.

The rate of “very satisfied” participants was higher in the e-learning group (14/35, 40%; 95% CI 24%-56%) versus the video group (4/26, 15%; 95% CI 5%-25%, *P*=.02) (Figure 12).

**Table 2:** Quiz results.

Item		Video group (n=34)	E-Learning group (n=41)	*P* value
Overall score, mean (SD)		35 (3)	38 (3)	<.001
Overall score, 95% CI		34-36	37-39	N/A^a^
**Detailed results by item, median (Q1-Q3)**				
	Key NIHSS^b^ concepts	5 (4-5)	5 (5-5)	.02
	Consciousness – Global	2 (2-2)	3 (2-3)	<.001
	Consciousness – Questions	3 (2-3)	3 (3-3)	.70
	Consciousness – Commands	2 (2-3)	3 (2-3)	.06
	Gaze	2 (2-3)	3 (2-3)	.34
	Visual	2 (2-2)	2 (2-2)	.23
	Facial Palsy	1 (0-2)	2 (1-2)	.04
	Motor arm	4 (4-5)	5 (4-5)	.17
	Motor leg	5 (4-6)	5 (4-5)	.23
	Ataxia	1 (1-1)	1 (1-2)	.03
	Sensory	3 (2-3)	3 (3-3)	.04
	Language	1 (1-2)	1 (1-1)	.63
	Dysarthria	2 (2-2)	2 (2-2)	.07
	Extinction and inattention	2 (2-3)	2 (2-3)	.14

^a^N/A: not applicable.

^b^NIHSS: National Institutes of Health Stroke Scale.

**Figure 13:**
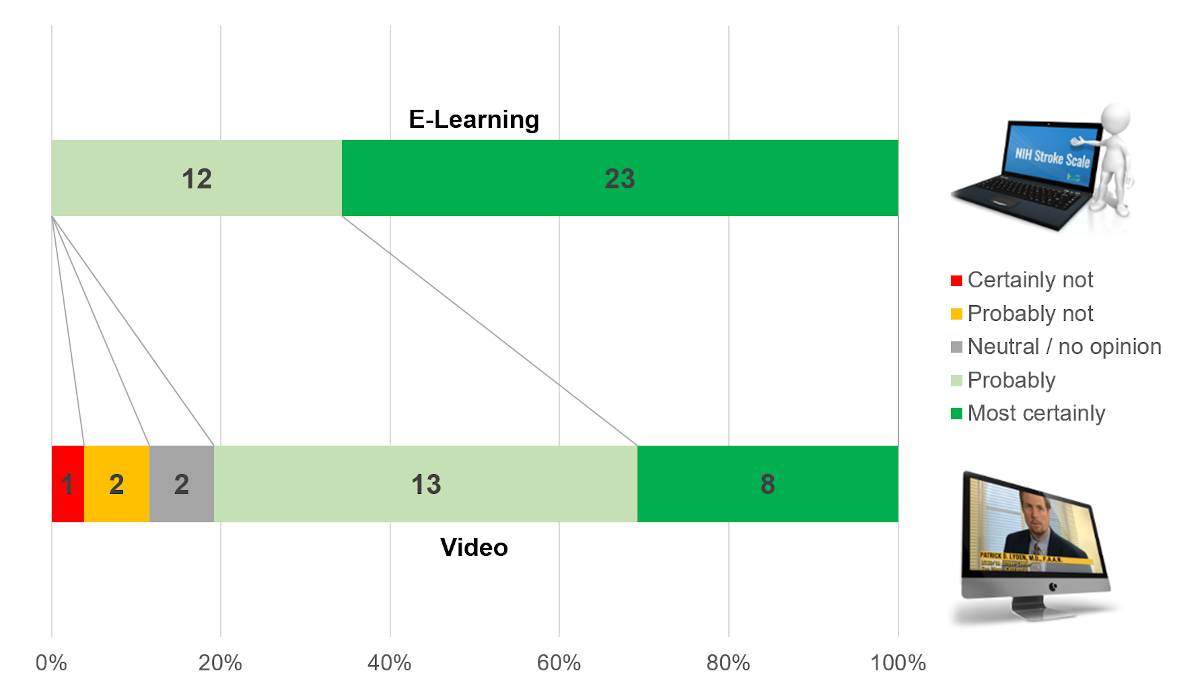
Likelihood that the participants in the e-learning and video groups would recommend the course.

Although the precise total learning time dedicated to either method could not be assessed due to the study design, no statistical difference regarding the perceived duration of the course was identified (80% (28/35) adequate in the e-learning group, 95% CI 67%-93%, vs 65% (17/26) 95% CI 47%-83%, *P*=.25). There was no significant difference regarding the perceived difficulty of the course, as 84% (16/19) (95% CI 68%-100%) found it “easy or very easy” in the e-learning group versus 53% (8/15) (95% CI 28%-78%) in the video group (*P*=.07). Participants who followed the e-learning method were more likely to recommend it to a colleague; 23/35 participants (66%) answered “Yes, most certainly” (95% CI 50%-82%), versus 8/26 (31%; 95% CI 13%-49%) in the video group, *P*=.007 (Figure 13). The proportions of participants in the two groups who followed the course over less than 12 hours were similar (58% (14/24) in the e-learning group, 95% CI 38%-78%, versus 52% (17/33) in the video group, 95% CI 35 to 69, *P*=.79).

The post hoc calculation showed a power of 97%. None of the 4 preplanned sensitivity analyses showed any major changes in the direction of the effect. The multivariable linear regression only showed a minor change in the coefficient (<15%), confirming these results.

## Discussion

### Principal Findings

In this study, asynchronous distance learning using a highly interactive e-learning module yielded better results than following the traditional didactic video on the web. The superiority of a previous version of this module has already been established in Swiss paramedics following an onsite computer-based course [23]. The present study confirms the generalizability of these findings when using this method for asynchronous distance learning in a different population of learners, namely, fifth year medical students. Indeed, although paramedics follow a 3-year curriculum focusing on critical emergencies, baseline knowledge and understanding of neurosciences should be higher in students on the verge of obtaining their master of medicine degree [34]. This assumption is supported by the median score of the control group, which was higher by two points in this study than it was in paramedics [23].

The shortcomings we had identified in the previous iteration of the e-learning module seem to have been addressed, as embedding cutscenes from the original video into every chapter of the module improved the impact of the module on knowledge acquisition. The use of short videos associated with active learning activities such as guiding questions or interactive elements has been shown to enhance knowledge acquisition and retention [35]. Interactivity itself is also known to improve both engagement and performance in medical students [36,37].

Slightly less than half of all potential participants completed their allocated learning path. Considering that the learning material was optional and that students’ summative assessments of this semester were replaced by formative assessments, the participation rate is rather encouraging given the global lack of incentive. More encouraging still is the proportion of students who would recommend the course to their peers, as such mechanisms may increase students' involvement [38]. As many medical students actively helped on the front lines during the crisis, some of them may have been prevented from participating in this study owing to their high workload [39].

The quiz shown to the participants upon completion of the learning material included not only the full evaluation of 3 different stroke patients, but also 5 general questions we had designed Multimedia Appendix 2. While this could be considered as a potential bias in the study design, these questions were solely linked to key elements and basic principles of the NIHSS, and their understanding is essential to the correct application of the scale Multimedia Appendix 2. Our aim was indeed to evaluate whether knowledge acquisition was different when presenting similar content in different learning formats. As the overall score regarding these questions was high in both groups, and as other significant results favored the e-learning method, there is little probability that these 5 initial questions induced a bias.

In many hospitals, the NIHSS is commonly used to triage stroke victims and help reduce both door-to-CT (CT: computed tomography) and door-to-needle times [9]. Decreasing these times is associated with better neurocognitive and functional outcomes [40]. Moreover, the adoption of a common scale between different specialists seems necessary to improve reproducibility and avoid the misinterpretation that can result from the use of different scores [41]. Swift acquisition and mastery of the NIHSS is therefore an essential skill for medical students, as most will be required to take care of stroke patients during their residency while working in the emergency department or in the neurology department. This is further strengthened by the fact that medical students often perceive neurology as the most difficult medical discipline, and the development of negative perceptions toward this specialty could lead to avoidance mechanisms when considering a career or treating a patient [42]. We might therefore assume that any kind of stroke-directed educational program could help raise awareness in non-neurologist physicians and thus increase the rate of correct treatment while decreasing door-to-needle time.

### Limitations and Strengths

This study has limitations that must be acknowledged. The main limitation is that we only measured immediate knowledge acquisition; we were unable to assess knowledge retention due to the study design and the limited timeframe. As this latter parameter is critical to the clinical application of the NIHSS, further studies will be needed to assess whether the e-learning method improves retention and leads to more accurate application of the scale. Moreover, the precise time taken to complete either learning method was not evaluated in this study. While it can be argued that dedicating more time to learning given content should yield better results, studies have shown that engagement is the most important factor regarding knowledge acquisition [43]. Although time to learning material completion is an interesting outcome, we chose not to record these data for two main reasons: risk of unblinding and technical limitations. As the time required to watch the video is fixed unless the participants elect to use the video commands, and as most of them chose not to use this option in a previous study [23], we thought it better not to risk unnecessarily unblinding the data analyst. The technical aspect was linked to the web-based learning management system and to the mode of delivery of the teaching material. As access to the university premises was barred during the study period, participants followed the learning material from many different locations. Interruptions in the learning process may therefore have occurred; however, we had no means of recording recurrent short breaks in the group that followed the e-learning module, as pauses may also have resulted from taking notes, mulling over the content, or simply rereading some of the written paragraphs to better understand them. To mitigate this limitation, a sensitivity analysis comparing participants who completed the study path in less and more than 12 hours was performed. Reassuringly, no difference was noted.

Despite these limitations, this study also has several strengths, including the randomization, the blinding mechanisms, the electronic data acquisition, the originality of the learning method and its mode of delivery in the context of the COVID-19 pandemic.

### Conclusion

Compared to the traditional didactic video, a highly interactive e-learning module enhances distant NIHSS knowledge acquisition in medical students.

## Appendix

Multimedia Appendix 1 Mailing template.

Multimedia Appendix 2 Original questions used in the 50-question quiz.

Multimedia Appendix 3 CONSORT-eHEALTH checklist (V 1.6.1).

## Abbreviations

CHERRIES: Checklist for Reporting Results of Internet E-Surveys
CSV: comma-separated values
CT: computed tomography
NIHSS: National Institutes of Health Stroke Scale
UGFM: University of Geneva Faculty of Medicine
