# Supplementary material for: Asynchronous Distance Learning of the National Institutes of Health Stroke Scale During the COVID-19 Pandemic (E-Learning vs Video): Randomized Controlled Trial
Source: J Med Internet Res. 2021 Jan 15;23(1):e23594. doi: 10.2196/23594 (PMC7812917; doi:10.2196/23594)
Supplement: Multimedia Appendix 1 [file jmir_v23i1e23594_app1.pdf]

Cher.e étudiant.e,

Dans le cadre des AMC de 2ème section, vous êtes invité à participer à une étude évaluant deux méthodes d'enseignement à distance. Cette étude a reçu l'aval de la Commission Cantonale d'Éthique de la Recherche.

Votre participation est bien entendu sur base volontaire. Cependant, le comité Master et les responsables de l'AMC de neurologie-neurochirurgie soutiennent cette étude et vous encouragent à y participer. Les activités qu'elle propose représentent en effet une opportunité d'apprentissage complémentaire au curriculum planifié pour la neurologie, bienvenue dans le contexte actuel.

Comme vous le savez, l'échelle NIHSS (National Institutes of Heath Stroke Scale) est la référence employée pour évaluer les patient.e.s victimes d'AVC. Plusieurs méthodes d'enseignement de cette échelle ont été décrites, l'une des plus connues étant une vidéo développée par le Professeur Patrick Lyden. Des médecins des HUG ont développé un module e-learning se basant notamment sur cette vidéo. Nous avons besoin de votre participation pour valider l'emploi de ce module dans le cadre de l'enseignement en médecine !

Comme évoqué en préambule cette participation vous sera utile car vous apprendrez l'utilisation de l'échelle et reverrez les éléments détaillés du status neurologique à effectuer en cas d'AVC. Cet apprentissage vous sera également utile pour votre examen, qui, même s'il n'est pas sanctionnel, restera un indicateur important de vos connaissances et compétences.

Si vous acceptez de participer, vous serez aléatoirement attribués.es à un des deux groupes suivants: groupe vidéo ou groupe e-learning. Nous n'avons aucun moyen de connaître votre allocation. Vous commencerez par répondre à quelques questions (moins de 1 minute), avant d'être invité.e à suivre l'une des deux méthodes de formation (en fonction de votre allocation). Dès la formation terminée, un quiz de 50 questions vous sera proposé. A la fin du quiz vous recevrez un feedback. Votre score vous sera donné, et vous pourrez passer en revue toutes les questions, voir les réponses attendues ainsi que vos propres réponses. Dès cette revue terminée, une dernière série de questions courtes sur votre appréciation de la méthode d'enseignement vous sera proposée (< 1 minute). Ensuite vous aurez librement accès à tout le matériel de formation, vidéo et e-learning, et ce jusqu'à votre examen prévu en juin.

Le temps consacré à cet apprentissage est estimé entre 2 et 4 heures (en fonction de votre rapidité). Il ne s'agit en aucun cas d'une épreuve de vitesse, et aucune limite de temps ne vous est imposée, si ce n'est d'avoir terminé le parcours avant la date de l'examen.

Votre confidentialité est bien entendu garantie: les créateurs de la plateforme d'étude/formation n'ont pas accès à votre adresse e-mail, et les membres de la faculté n'ont pas d'accès direct aux données recueillies sur la plateforme. Il ne sera donc pas possible de lier votre identité à vos réponses, et aucune question permettant de vous identifier ne sera posée durant votre parcours.

Des informations supplémentaires se trouvent directement sur la plateforme, voici les informations pour vous connecter :

Adresse internet (URL) de la plateforme d'étude/formation: https://nihss-etumed.cardiomobile.ch
Nom d'utilisateur: {username}
Mot de passe: {password}

Nous vous remercions de l'attention que vous portez à cette invitation et restons à votre disposition en cas de question.

Meilleures salutations

Signature(s)

Professeur G Savoldelli, Président du comité Master
Professeur F Assal, Responsable de l'AMC de neurologie
Dr L Suppan, Responsable de l'étude
