# Supplementary material for: Asynchronous Distance Learning of the National Institutes of Health Stroke Scale During the COVID-19 Pandemic (E-Learning vs Video): Randomized Controlled Trial
Source: J Med Internet Res. 2021 Jan 15;23(1):e23594. doi: 10.2196/23594 (PMC7812917; doi:10.2196/23594)
Supplement: Multimedia Appendix 2 [file jmir_v23i1e23594_app2.pdf]

Supplementary Table 1

Original questions used in the quiz

Original question in French	English translation
Lorsque l'on calcule le score NIHSS, pour la motricité des jambes:<br>• Chaque jambe est testée, et le meilleur score est retenu<br>• Chaque jambe est testée, et le moins bon score est retenu<br>• Chaque jambe est testée, et il faut calculer la moyenne<br>• Chaque jambe est testée séparément, et chacun des scores obtenus est pris en compte	***When calculating the NIHSS "Leg Motor" item:***<br>• Each leg is tested, and the best score is taken into account<br>• Each leg is tested, and the worst score is taken into account<br>• Each leg is tested, and the average must be calculated<br>• Each leg is tested separately, and each of the scores obtained is taken into account
Lorsque l'on calcule le score NIHSS, pour la motricité des bras:<br>• Chaque bras est testé, et le meilleur score est retenu<br>• Chaque bras est testé, et le moins bon score est retenu<br>• Chaque bras est testé, et il faut calculer la moyenne<br>• Chaque bras est testé séparément, et chacun des scores obtenus est pris en compte	***When calculating the NIHSS "Arm Motor" item:***<br>• Each arm is tested, and the best score is taken into account<br>• Each arm is tested, and the worst score is taken into account<br>• Each arm is tested, and the average must be calculated<br>• Each arm is tested separately, and each of the scores obtained is taken into account
Lorsque l'on effectue le score NIHSS:<br>• "0" est toujours le meilleur score<br>• "3" est toujours le meilleur score<br>• "NT" peut être codé pour tous les éléments testés<br>• Il n'est pas utile de détailler la raison pour laquelle "NT" est codé	***When performing the NIHSS score:***<br>• "0" is always the best score<br>• "3" is always the best score<br>• "NT" can be coded for all elements tested<br>• It is not useful to give details when "NT" has been coded
La mention "NT":<br>• Peut être codée pour tous les éléments testés dans le NIHSS<br>• Doit obligatoirement être accompagnée d'un justificatif<br>• Ne s'applique qu'aux patients intubés<br>• Ne s'applique que pour la motricité des bras et des jambes	***The "NT" score:***<br>• Can be coded for all NIHSS items<br>• Must be accompanied by a detailed explanation<br>• Can only be coded if the patient is intubated<br>• Can only be coded for the "Leg Motor" and "Arm Motor" items
Le score NIHSS:<br>• Correspond à un examen neurologique standardisé<br>• Permet un suivi temporel de l'état neurologique des patients<br>• Est reproductible (plusieurs observateurs différents obtiendront le même score pour un même patient à un même moment)<br>• Toutes ces réponses sont justes	***The NIHSS score:***<br>• Is a standardized neurological examination<br>• Allows monitoring of the neurological status of patients over time<br>• Is reproducible (several different observers will obtain the same score for the same patient at the same time)<br>• All of the above

Correct answers are colored in green
